# *OsDDM1b* Controls Grain Size by Influencing Cell Cycling and Regulating Homeostasis and Signaling of Brassinosteroid in Rice

**DOI:** 10.3389/fpls.2022.873993

**Published:** 2022-04-08

**Authors:** Mingliang Guo, Wenchao Zhang, Mohammad Aqa Mohammadi, Zhimei He, Zeyuan She, Maokai Yan, Chao Shi, Lingwei Lin, Aqiong Wang, Jindian Liu, Dagang Tian, Heming Zhao, Yuan Qin

**Affiliations:** ^1^State Key Laboratory of Ecological Pest Control for Fujian and Taiwan Crops, College of Plant Protection, Fujian Agriculture and Forestry University, Fuzhou, China; ^2^College of Agriculture, Fujian Agriculture and Forestry University, Fuzhou, China; ^3^College of Horticulture, Fujian Agriculture and Forestry University, Fuzhou, China; ^4^State Key Laboratory for Conservation and Utilization of Subtropical Agro-Bioresources, Guangxi Key Lab of Sugarcane Biology, College of Agriculture, Guangxi University, Nanning, China; ^5^Fujian Provincial Key Laboratory of Haixia Applied Plant Systems Biology, College of Life Sciences, Fujian Agriculture and Forestry University, Fuzhou, China; ^6^Biotechnology Research Institute, Fujian Provincial Key Laboratory of Genetic Engineering for Agriculture, Fujian Academy of Agricultural Sciences, Fuzhou, China; ^7^Center for Crop Biotechnology, College of Agriculture, Anhui Science and Technology University, Fengyang, China

**Keywords:** rice, Snf2 family, *OsDDM1b*, grain size, cell proliferation, brassinosteroid

## Abstract

Snf2 family proteins are the crucial subunits of chromatin-remodeling complexes (CRCs), which contributes to the biological processes of transcription, replication, and DNA repair using ATP as energy. Some CRC subunits have been confirmed to be the critical regulators in various aspects of plant growth and development and in epigenetic mechanisms such as histone modification, DNA methylation, and histone variants. However, the functions of Snf2 family genes in rice were poorly investigated. In this study, the relative expression profile of 40 members of Snf2 family in rice was studied at certain developmental stages of seed. Our results revealed that *OsCHR741/OsDDM1b* (*Decrease in DNA methylation 1*) was accumulated highly in the early developmental stage of seeds. We further analyzed the *OsDDM1b* T-DNA insertion loss-of-function of mutant, which exhibited dwarfism, smaller organ size, and shorter and wider grain size than the wild type (*Hwayoung*, HY), yet no difference in 1,000-grain weight. Consistent with the grain size, the outer parenchyma cell layers of lemma in *osddm1b* developed more cells with decreased size. *OsDDM1b* encoded a nucleus, membrane-localized protein and was distributed predominately in young spikelets and seeds, asserting its role in grain size. Meanwhile, the *osddm1b* was less sensitive to brassinosteroids (BRs) while the endogenous BR levels increased. We detected changes in the expression levels of the BR signaling pathway and feedback-inhibited genes with and without exogenous BR application, and the alterations of expression were also observed in grain size-related genes in the *osddm1b*. Altogether, our results suggest that *OsDDM1b* plays a crucial role in grain size *via* influencing cell proliferation and regulating BR signaling and homeostasis.

## Introduction

In eukaryotes, genomic DNA is wrapped around the histone octamer to form a nucleosome which is the subunit of chromatin. A highly organized chromatin structure is essential for gene expression in eukaryotes. Chromatin packages large quantities of genetic information into the nucleus and provides an efficient integrative platform that translates signals to regulate gene responses (Hu et al., 2013; Zhang et al., 2019). The mechanism of epigenetic regulation involves histone modification, histone variants, DNA methylation, and chromatin remodeling (Hu et al., 2013). A recent study reveals that many proteins have been identified to mediate these processes, among which Snf2 family proteins are responsible for chromatin remodeling to regulate gene expression using ATP energy (Cairns, 2005; Hu et al., 2013; Zhang et al., 2019). According to the helicase-like region, the Snf2 family proteins are classified into six groups with two conserved domains (SNF2_N and Helicase_C) (Andrew et al., 2006). Each group could be subdivided into 24 subfamilies, and some of these subfamilies are unique for specific organisms and others are ubiquitous (Andrew et al., 2006; Song et al., 2021).

Snf2 family proteins have been identified to contain 41, 45, and 40 members in *Arabidopsis*, tomato, and rice, respectively. Many of these proteins have been reported to play the essential roles in regulating plant development and stress responses (Knizewski et al., 2008). For example, the loss-of-function of *SPLAYED* (*SYD*) causes shoot apical meristem (SAM) defects in *Arabidopsis*; SYD interacts with WUSCHEL (WUS), the central regulator in SAM (Kwon et al., 2005); mutations of *BRM* cause reduced root length and plant size (Farrona et al., 2004), curly leaves (Hurtado et al., 2006), sensitive to abscisic acid (ABA) (Han et al., 2012), and early flowering (Farrona et al., 2011). Indeed, BRM is always recruited and associated with other nuclear proteins, such as RELATIVE OF EARLY FLOWERING 6 (REF6) and FORGETTER1 (FGT1), to modulate gene transcription (Brzezinka et al., 2016; Li et al., 2016). BRM also interacts with TEOSINTE BRANCHED1 CYCLOIDEA AND PCF-CODING GENE (TCP4), ANGUSTIFOLIA3 (AN3), and BREVIPEDICELLUS (BP) to regulate leaf development and inflorescence architecture (Efroni et al., 2013; Vercruyssen et al., 2014) and PHY-INTERACTING FACTOR1 (PIF1) to modulate *PROTOCHLOROPHYLLIDE OXIDOREDUCTASE C* (*PROC*) expression for chlorophyll biosynthesis (Zhang et al., 2017). The plants overexpressing *AtCHR12* showed growth arrest of primary buds and stem under drought and heat stresses (Mlynarova et al., 2007). Moreover, both DEFECTIVE IN RNA-DIRECTED DNA METHYLATION 1 (DRD1, a DRD1 subfamily member) and DECREASED DNA METHYLATION 1 (DDM1, an Lsh subfamily member) are involved in DNA methylation process (Jeddeloh et al., 1999; Kanno et al., 2004). In addition, the mutant of *DRD1* and *DDM1* exhibits the delay of leaf senescence (Cho et al., 2016). Furthermore, PHOTOPERIOD-INDEPENDENT EARLY FLOWERING 1 (PIE1, an Swr1 subfamily member) is responsible for H2A.Z deposition to regulate flowering and plant development (Choi et al., 2007), and PICKLE (a Mi-2 subfamily member) regulates the H3K27me3-enriched genes (Zhang et al., 2012) and the plant hormone signaling, such as brassinosteroid (BR), gibberellic acid (GA), and cytokinin (CK) (Kakimoto and Physiology, 2011; Zhang et al., 2014). CHROMATIN REMODELING 4 (CHR4) interacts with transcription factors and affects the expression of critical floral regulators to mediate the flowering response pathways of inflorescence meristem to promote floral identity (Sang et al., 2020).

In addition to *Arabidopsis*, several Snf2 family proteins have been identified and studied in other plant species. For example, constitutively, *SlCHR1* (*Solyc01g079690*) overexpression causes reduced plant growth (Folta et al., 2016). *DRD1* and Snf2 subfamilies are involved in stress responses in tomato plants (Bargsten et al., 2013). In rice (*Oryza sativa* L.), the loss-of-function of *CHR729* (a member of Mi-2 subfamily) results in dwarf, later flowering, less tiller, and narrow leaf by affecting contents of GA_3_ (Hu et al., 2012; Ma et al., 2015). *OsALT1* (*alkaline tolerance 1*, *OsCHR706*), an Snf2 family chromatin remodeling ATPase, negatively regulates alkaline tolerance (Guo et al., 2014), and *OsBRHIS1* plays a critical role in SA-independent disease resistance by suppressing innate immunity (Li et al., 2015).

*OsDDM1a* and *OsDDM1b*, as the homologous genes to *Arabidopsis DDM1*, are involved in DNA methylation (Higo et al., 2012), which results in high methylation levels at CHG and CG contexts (Tan et al., 2016). The mutation of *OsDDM1* decreases histone H3K9me2 and increases the small heterochromatic RNA and long non-coding RNA (Tan et al., 2018). However, the relative expression profile of Snf2 gene family in rice during reproductive development has not been systematically analyzed. Here, we deeply investigated the expression profiles of the rice Snf2 genes during different developmental stages of seed and characterized the phenotype of *osddm1b*. The mutant displayed dwarfism and shorter and wider grain size and was insensitive to BR treatments. Our results provide an in-depth knowledge for further exploration of the function of Snf2 family proteins in rice, which indicates that *OsDDM1b* helps to regulate organ size by influencing cell proliferation and involving in BR response regulation.

## Materials and Methods

### Plant Material and Growth Condition

The *OsDDM1b* T-DNA insertion mutant (PFG_2B-60109) and wild-type cultivar *Hwayoung* were obtained from the Crop Biotech Institute, Department of Plant Systems Biotech, Kyung Hee University. The T-DNA information was obtained from SIGnAL database at http://signal.salk.edu/cgi-bin/RiceGE. The plants were grown in the greenhouse at 22–32°C and 80–90% humidity with a 14-h/10-h (light/dark) photoperiod. About 1, 3, 7, 10, and 25 days after fertilization (DAF) of seeds were harvested using micro-dissection needles, frozen in liquid nitrogen immediately, and stored at -80°C for total RNA extraction. A total of three biological replicates of each sample were collected for experimental analysis.

The T-DNA insertion in *osddm1b* was confirmed by PCR using the primers 2B-60109-Lp and 2B-60109-Rp and the T-DNA-specific primer Rb (2707). The full-length coding sequence of *OsDDM1b* was amplified from WT cDNA by PCR and cloned into the *Eco*RI/*Kpn*I sites of the pCAMBIA2300 vector. The derived constructs were introduced into the *osddm1b* by *Agrobacterium tumefaciens*-mediated transformation. The relative expression levels of *OsDDM1b* were detected in 7-day-old seedling leaves of *osddm1b* and complementary mutants by the primers P1 and P2. The primers sequences are described in Supplementary Table 2 for genotyping identification. The gene structure of *OsDDM1b* was searched from Gene Structure Display Server.^1^

### RNA Isolation and qRT-PCR Analysis

Total RNAs of all collected samples were extracted using Plant RNeasy Mini kit (Qiagen, Hilden, Germany). About 1 μg RNA was reverse-transcribed using the PrimeScript RT-PCR kit (Takara, Kyoto, Japan) according to the manufacturer’s instruction (Cai et al., 2019). The relative expression level was detected by qRT-PCR using the Bio-Rad qRT-PCR system (Foster City, CA, United States) and SYBR Premix Ex TaqII (TaKaRa Perfect Real Time) (Zhang et al., 2020). The qRT-PCR program was 95°C for 30 s; 40 cycles of annealing at 95°C for 5 s and extension at 60°C for 35 s; and 95°C for 15 s (Zhang et al., 2020; Zhao et al., 2021). The rice *OsUBQ5* gene was used as an internal control. To evaluate the relative expression levels of the examined genes, we used the comparative *C*_*T*_ method (Su et al., 2017). The gene-specific primers are listed in Supplementary Tables 3–6.

### Observation of Pollen and Ovule in Rice

Several anthers were randomly selected before pollination and placed on microscopic slide for dissecting with the micro-dissection needles using a microscope. The pollen was stained with 1% I_2_-KI solution and observed under the microscope (Ren et al., 2019). For the analysis of the ovule fertility, the spikelets before pollination were fixed in the FAA solution (50% ethanol: acetic acid: formaldehyde = 89:6:5) and using the whole-mount eosin B-staining confocal laser scanning microscopy (WECLSM) to observe the ovule development (Zeng et al., 2007, 2009). After fixation, eosin B staining, and clearing, the ovaries were divided from spikelets and observed using a Leica SP8 CLSM (Leica Microsystems) to screen the ovule (Zhao et al., 2020).

### Promoter Fusion and GUS Staining

A 2,559-bp fragment upstream of *OsDDM1b* ATG codon sequence was amplified by PCR from DNA of rice leaf using the primers listed in Supplementary Table 3. Then, the products were constructed into a pENTER/D-TOPO vector (Invitrogen, CA, United States). After that, the positive clones were recombined with the pGWB533 vector by LR Clonase II enzyme (Invitrogen, CA, United States). The wild-type ZH11 (Zhonghua 11) callus was transformed using the *Agrobacterium*-mediated transformation using the *pOsDDM1b*: green fluorescent protein (GUS) recombinant construction. The transgenic plant tissues were incubated in β-glucuronidase (GUS) staining buffer overnight at 37°C (Jefferson et al., 1987) and dehydrated in an ethanol series to remove the chlorophyll (Jiang et al., 2012). The images were viewed under a Leica (M205 FA) microscope.

### Subcellular Localization of OsDDM1b

A 2,547-bp segment of *OsDDM1b* coding sequence was amplified from WT cDNA using the primers listed in Supplementary Table 3. The PCR fragments were cloned into the pENTER/D-TOPO vector (Invitrogen, CA, United States), and pENTER/D-TOPO clones were recombined into the pGWB505 vector using LR Clonase II enzyme (Invitrogen, CA, United States). The 35S:*OsDDM1b*-GFP recombinant construction and 35S: GFP (vector control) were transformed into *Agrobacterium tumefaciens* (GV3101) and infiltrated with ER-mCherry marker to tobacco leaves. The fluorescence signals were observed using a confocal microscope (SP8, Leica, Germany), and the excitation wavelength was 488 nm.

### Histological Observation and Scanning Electron Microscope

The spikelets were fixed in FAA (50% ethanol, 5% acetic acid, and 3.7% formaldehyde) at 4°C overnight and dehydrated in a series of ethanol for observing the spikelet cell size and number. After fixing with chloroform, the samples were embedded in Paraplast Plus (Sigma). The samples were sliced into the 8-μm-thickness samples using an RM2245 rotary microtome (Leica). Sections were dewaxed in xylene and gradually rehydrated and dehydrated before staining with toluidine blue for light microscopy. For scanning electron microscope (SEM), fresh materials were applied directly to the scanning electron microscope (Ren et al., 2018; Yu et al., 2020). The cell number and cell size in the outer parenchyma layer of the spikelet hulls were measured by ImageJ^2^ (Yu et al., 2020).

### BR Treatment

For the lamina joint test, the seeds were germinated in water at 37°C and grown hydroponically in a nutrient solution containing 0, 0.01, 0.1, and 1 μm epi-BL for 1 week. Then, the second leaf lamina joint was measured. For the coleoptile elongation assay, the seeds were sterilized with 10% hypochlorous acid and germinated under dark conditions in half-strength MS medium supplemented with 0, 0.01, 0.1, and 1 μm epi-BL. Coleoptile length were measured after a week (Liu S. Y. et al., 2015).

### Measuring Endogenous BRs

Plants were grown hydroponically in nutrient solution for 10 days. The shoots and roots (equivalent to 1 g of fresh weight) of wild type and *osddm1b* were harvested and ground with phosphate-buffered saline solution immediately. BR endogenous contents were analyzed using the BR ELISA kit according to the manufacturer’s protocol (SINOBESTBIO, Shanghai, China) by UV spectrophotometer in 450 nm.

## Results

### The Comparative Expression Level of Snf2 Gene Family During Seed Development

**FIGURE 1:**
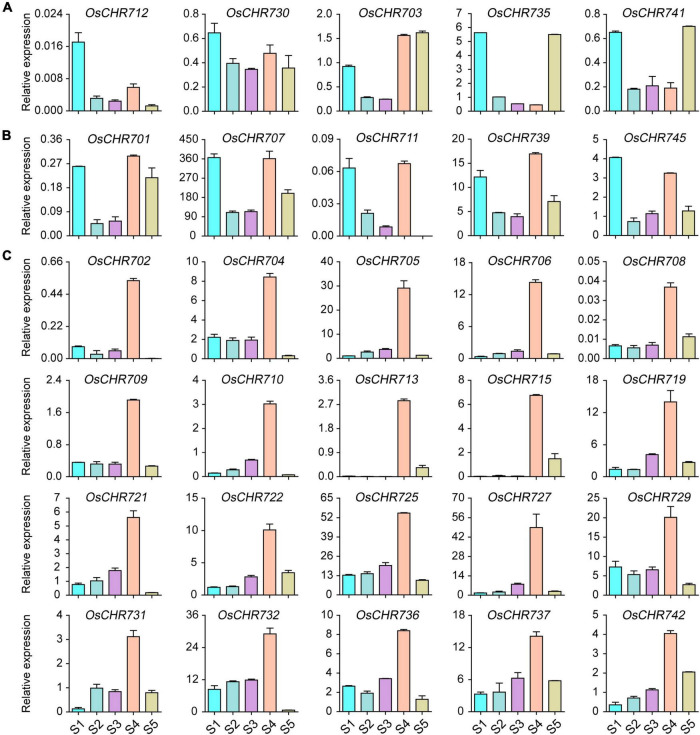
The relative expression level of Snf2 gene family in the wild-type (ZH11) seeds. **(A)** Preferentially expressed Snf2 genes in the seeds’ early or late developmental stages. **(B)** Predominantly expressed Snf2 genes in the seeds’ early and mid-term developmental stages. **(C)** Explicitly expressed Snf2 genes in the seeds mid-term developmental stages. S1, the seeds of 1 DAF; S2, the seeds of 3 DAF; S3, the seeds of 7 DAF; S4, the seeds of 10 DAF; S5, the seeds of 25 DAF.

The development of seeds is important to get high yield, and many genes highly regulate the developmental process. To explore the expression level of the Snf2 family genes during seed development, we collected the seeds from 1, 3, 7, 10, and 25 DAF. We checked the gene expression level shown in Figure 1 and Supplementary Figure 1. The results showed that *OsCHR712* and *OsCHR730* were predominantly expressed in early developmental stages of seeds (S1), and *OsCHR703*, *OsCHR735*, and *OsCHR741* were preferentially expressed in both S1 and S5 stages (Figure 1A). In addition, *OsCHR701*, *OsCHR707*, *OsCHR711*, *OsCHR739*, and *OsCHR745* were preferentially expressed in both S1 and S4 stages (Figure 1B). There were 23 Snf2 genes that showed similar expression pattern which were predominantly expressed in the S4 stage, such as *OsCHR702*, *OsCHR704*, *OsCHR705*, *OsCHR706*, *OsCHR708*, *OsCHR709*, *OsCHR710*, *OsCHR713*, *OsCHR715*, *OsCHR719*, *OsCHR721*, *OsCHR722*, *OsCHR725*, *OsCHR727*, *OsCHR729*, *OsCHR731*, *OsCHR732*, *OsCHR736*, *OsCHR737*, *OsCHR742* (Figure 1C), *OsCHR720*, *OsCHR726*, and *OsCHR740* (Supplementary Figure 1A). These results indicated that Snf2, a sizeable family that exhibits specific expression patterns, may have unique functions in different developmental stages of seed in rice.

### Characterization of the *OsDDM1b* Mutant

**FIGURE 2:**
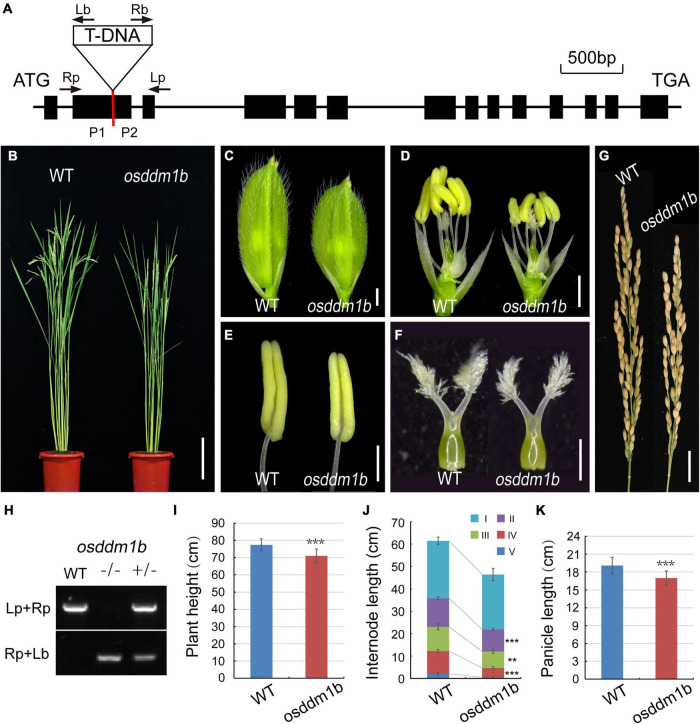
Morphology analysis of the wild type (*Hwayoung*, HY) and *osddm1b*. **(A)** The gene structure of *OsDDM1b* and the triangle indicate the T-DNA insertion site. The arrows show the primers which were used in the genotyping study, and P1 and P2 are the positions of primers using qRT-PCR. **(B)** The phenotype of wild type (left, HY) and *osddm1b* (right) plants at maturity. Bar = 15 cm. **(C,D)** The spikelets of wild type (HY) and *osddm1b*. Bar = 1 mm. **(E,F)** Anthers and pistils of wild type **(left)** (HY) and *osddm1b* **(right)**. Bar = 500 μm, 250 μm. **(G)** Panicles of wild type (HY) and *osddm1b*. Bar = 2 cm. **(H)** Genotyping of T-DNA insertion plants. **(I–K)** The average plant height, internode length, panicles length of wild type (HY), and *osddm1b*, respectively (*n* = 20 per sample). Data are given as means ± SD. **p < 0.01, ***p < 0.001 compared with wild type (HY) using Student’s *t*-test.

The previous studies showed that two homologous genes in rice (*Oryza sativa* var. Nipponbare) offered 60% identity to *DDM1* (AT5G66750) (Higo et al., 2012). The two genes, *OsDDM1a* (LOC_Os09g27060) and *OsDDM1b* (LOC_Os03g51230), share 93% amino acid identity to each other (Higo et al., 2012; Tan et al., 2016). Here, we identified a T-DNA insertion approximately 0.55 kb downstream of the translation start site ATG in *osddm1b* (2B-60109.L) from *Hwayoung* background (Figure 2A), which were identified in the SIGnAL database.^3^ The homozygous plants for the T-DNA insertion were identified and obtained by PCR (Figure 2H). In addition, the PCR products of homozygous plants were sequenced, and the sequencing results were aligned with the genome sequence (*OsDDM1b*) and vector sequence (2707), and then, we found the T-DNA insertion site (Supplementary Figure 3).

In *osddm1b*, the dwarfism was observed in the seedlings and mature plants (Figures 2B,I and Supplementary Figure 2A and Table 1). The length of first internode was similar to the wild type. However, the second, third, and fourth internodes of *osddm1b* were shortened, which suggests that abnormal internode elongation leads to dwarf phenotype (Figure 2J and Supplementary Figure 2B). The reproductive organs of the *osddm1b*, such as spikelets, anthers, and pistils, were much smaller than those of wild type (Figures 2C–F). The panicle morphology in *osddm1b* exhibited a significantly reduced growth of 11.03% than the wild type at mature stage (Figures 2G,K). Then, we compared male and female gametophyte morphology in the *osddm1b* and wild-type plants. The staining of pollen with iodine potassium iodide (I_2_-KI) solution and the observation of ovule with eosin B by confocal showed that the male (Supplementary Figures 2C–D) and female gametophytes (Supplementary Figures 2E,F) have no noticeable difference compared to the wild type. However, the seed setting in *osddm1b* was decreased to 84.39% (Supplementary Figure 2G).

**FIGURE 3:**
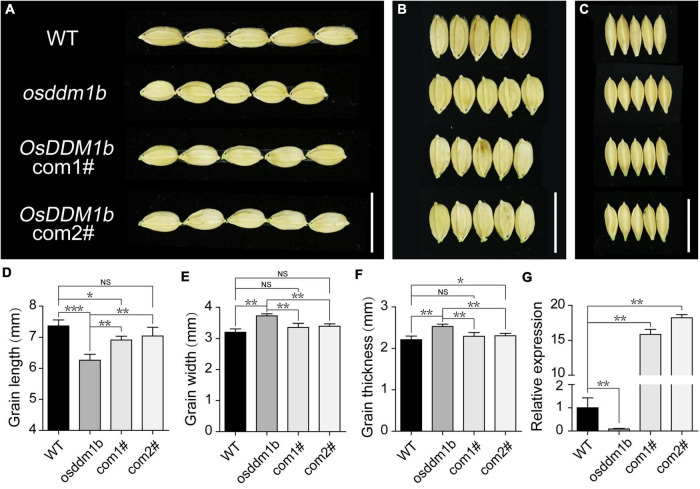
Functional verification of *OsDDM1b* in grain size. **(A–C)** Comparation on grain size of *osddm1b*, complementation mutants with wild type (*Hwayoung*, HY), respectively. Bar = 1 cm. **(D–F)** Grain length, width, and thickness of wild type (HY) and *osddm1b* (*n* = 60 per sample). **(G)** The relative expression levels of *OsDDM1b* in *osddm1b*, complementation mutants, and wild type (HY) in the leaves of 7-day-old seedlings. Data are given as means ± SD. ^NS^*p* > 0.05, *p < 0.05, **p < 0.01, ***p < 0.001 compared with wild type (HY) using Student’s *t*-test. NS: not significant.

To further confirm the effect of *OsDDM1b* on grain size, we examined the grain length, width, thickness, and 1,000-grain weight. The results showed that *osddm1b* had significantly reduced grain length of 8.15% than the wild type (Figures 3A,D, Supplementary Figure 2I,J), but it increased 17.59% in grain width (Figures 3B,E and Supplementary Figures 2K,M) and 10.67% in grain thickness, respectively (Figures 3C,F and Supplementary Figures 2L,N). Even though there was a change in the grain size, there was no noticeable change in the 1,000-grain weight (Supplementary Figure 2H). To verify the role of *OsDDM1b* in determining grain size in rice, we performed a genetic complementation test. An overexpression plasmid containing full-length coding sequence was inserted by the transformation into the *osddm1b*. Then, we got two positive complementary lines. The overexpression of *OsDDM1b* in *osddm1b* could partially complement the grain size defection of mutant (Figures 3A–C). The grain size (length, width, and thickness) was significantly different between the complementary lines and *osddm1b* (Figures 3D–F). Particularly, the grain width in the complementary lines was similar to wild type (Figure 3E). The *OsDDM1b* expression level was dramatically reduced in 7-day-old young leaves in *osddm1b* but increased in the complementary lines compared with those of the wild type (Figure 3G). The other defection phenotypes could be partially rescued in the complementation lines. This result indicated that the mutation in *OsDDM1b* was responsible for the phenotype of grain size exhibited in *osddm1b*.

### *OsDDM1b* Regulates Grain Size by Affecting Cell Proliferation

**FIGURE 4:**
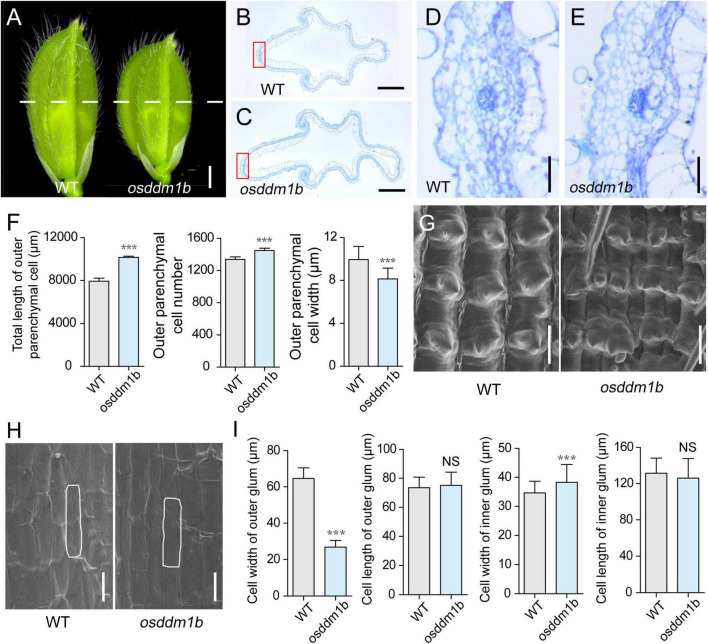
Histological analysis of spikelet hulls. **(A)** Young spikelet hulls of wild type (*Hwayoung*, HY) and *osddm1b*. The white line indicates the position of the cross-section. Bar = 1 mm. **(B,C)** Cross-sections of spikelet hulls from wild type (HY) and *osddm1b*, respectively. Bar = 500 μm. **(D,E)** Magnified view of the cross-section area boxed in panels **(B,C)**, respectively. Bar = 50 μm. **(F)** Statistical data of the total length, cell number, and length in the outer parenchyma layer (*n* = 12). **(G,H)** Scanning electron microscope (SEM) analysis of the outer and inner surfaces of glumes. Bar = 60 μm. **(I)** Statistical analysis of cell width and length in outer and inner glumes (*n* = 40, 60, respectively). Data are given as means ± SD. ***p < 0.001 compared with wild type (HY) using Student’s *t*-test.

The spikelet hullin *osddm1b* became shorter and more comprehensive compared with wild type before fertilization (Figure 4A). Since the cell division and/or cell expansion are responsible for the alteration in the final spikelet hull size and grain size, we carefully examined the cross-section of the central part of lemma and palea in mature spikelets and compared the epidermal cells of *osddm1b* and HY by scanning electron microscope. The hull cross-section of the spikelet hullin *osddm1b* revealed a significant increase of 27.84% in the length of the total cells and 8.15% in cell number of outer parenchyma in the *osddm1b*. In comparison, the cell width was decreased by 18.07% in the outer parenchyma cells (Figures 4B–F). It also changed the number of rows of specialized cells with a rigid wall in the upper epidermis (Supplementary Figures 4A–B). The data suggested that the cell division was significantly increased in a transverse direction in the *osddm1b* spikelet hulls. We also investigated the expression of genes involved in the cell cycle, such as *CYCA2.2*, *CYClaZm*, *E2F2*, *CYCB2*, *MCM3*, *MCM4*, and *MCM5*. We found that these genes were upregulated in young panicles of *osddm1b*, which suggests that the increased cell number might have resulted from the elevated expression of genes that promote cell proliferation (Supplementary Figure 4C). In addition, we observed the average length and width of longitudinal epidermal cells in outer and inner glumes of wild type and *osddm1b* by scanning electron microscopy. An apparent 58.43% decrease in cell width was observed in the outer glum, but 10.41% in cell width was increased in the inner glum. Then, there is no significant difference in outer and inner glumes’ cell length in *osddm1b* (Figures 4G–I). These observations suggest that *OsDDM1b* might promote latitudinal growth by increasing cell proliferation.

### OsDDM1b Localization and Expression Pattern

**FIGURE 5:**
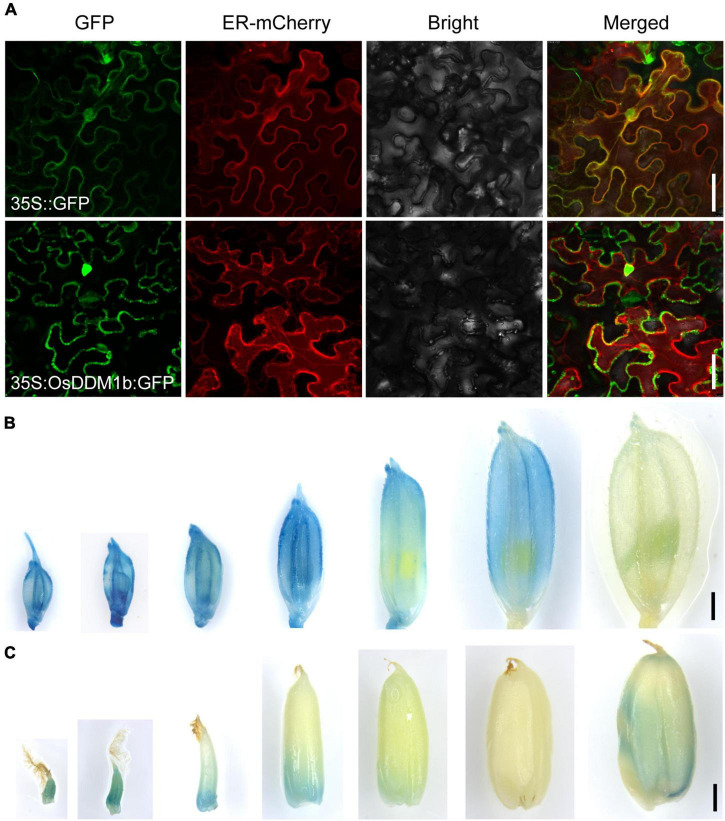
The expression profile of *OsDDM1b.* **(A)** Subcellular localization of OsDDM1b protein was shown to target the cytomembrane and nuclear by transient expression of 35:OsDDM1b-GFP in tobacco. The upper row is the 35S:GFP as the control. Bar = 50 μm. **(B,C)** Expression pattern detected in transgenic plants carrying the *pOsDDM1b*:GUS vector in ZH11 background. Developing spikelets in turn 1–2, 2–3, 3–4, 4–5, 5–6, 6–7 cm, and mature spikelet **(B)**; The seeds of 5 HAF and 1, 3, 7, 10, 15, and 25 DAF, respectively **(C)**. Bar = 1 mm. HAF, hours after fertilization; DAF, days after fertilization.

To investigate the subcellular localization of OsDDM1b, we performed a transient transformation assay. When *OsDDM1b*-green fluorescent protein (GFP) was transformed into tobacco leaves, the signals mainly targeted the nucleus and cell membrane (Figure 5A).

To further examine the expression profile of *OsDDM1b*, a construct was produced in which the *OsDDM1b* promoter (approximately 2.5 kb upstream of the ATG site) was fused to the β-glucuronidase gene (GUS) to transform wild-type (ZH11) plants. GUS signals were detected in the vegetative organs, such as root tips, young and mature leaf blades, stem, and internodes (Supplementary Figures 5A–D). We also detected at different developmental stages of the reproductive organs, such as spikelets, stamens, pistils, and seeds. GUS expression was visualized during the developmental stage of spikelet hulls except for the mature stage (Figure 5B). The developmental period of the spikelet can provide a reference for the pistil and stamen. Then, we estimated the developmental stage of pistil and stamen according to the length of the spikelet. Filaments of early stamens and the stamens of 4–5 mm and mature spikelets had the GUS signals (Supplementary Figure 5E). It was detected in the basal of pistils during the early stage (≤5 mm spikelet) and stigma of the mature stage (Supplementary Figure 5F). The GUS staining in seeds showed that it was highly expressed in the early stage [such as 5 h after fertilization (HAF), 1 DAF, 3 DAF, and 10 DAF] and mature seeds (Figure 5C), consistent with the results of the qRT-PCR. This expression profile indicated that *OsDDM1b* functioned in different young tissues, in line with the results presented above that *OsDDM1b* regulated cell division and proliferation rate.

### The *OsDDM1b* Mutant Is Less Sensitive to BRs

**FIGURE 6:**
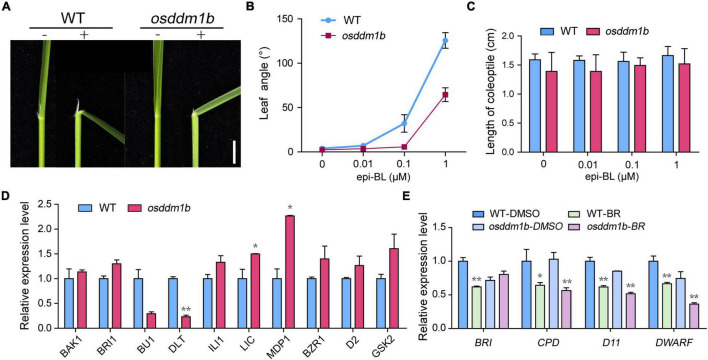
The *OsDDM1b* mutant is less sensitive to brassinolide (BL). Response to BR treatments, **(A)** Lamina joints of wild type (HY) and *osddm1b*. Bar = 2 mm. **(B)** Measurements of the lamina joint angle of wild type (HY) and *osddm1b* after various BR treatments. **(C)** Effect of BR on the coleoptile in wild-type (HY) and *osddm1b* seedling. **(D)** The expression level of BR signaling pathway-related genes and assayed using RNA from roots of 7-day-old seedlings cultured in half-strength MS medium. **(E)** Quantitative RT-PCR analysis of feedback-inhibited genes of BR biosynthesis. Data are given as means ± SD. **p < 0.01, *p < 0.05 compared with wild type (HY) using Student’s *t*-test.

The phenotype of *osddm1b* shown, such as erect and dark green leaves, dwarfism, and small grains, is typical BR response phenotypes in rice (Yamamuro et al., 2000; Hong et al., 2003). So, we hypothesize that *OsDDM1b* is involved in the BR response. We evaluated the sensitivity of *osddm1b* to 24-epibrassinolide (24-epiBL) using a lamina joint assay. As shown in the results, the second lamina of the wild type exhibited a 24-epiBL dosage-dependent inclination. By contrast, *osddm1b* did not show a blatant response (Figures 6A,B and Supplementary Figure 6A). The previous studies suggested that BR promoting coleoptile growth was an another indicator of a BR-responsive phenotype (Yamamuro et al., 2000; Li et al., 2002; Je et al., 2010). To analyze the response of coleoptiles to BR, we also performed a coleoptile elongation assay for BR sensitivity (Yamamuro et al., 2000). The wild-type and *osddm1b* seeds were germinated and grown under dark conditions in half MS medium containing different concentrations of 24-epiBL. The results showed that the relative elongation of coleoptile treatments was much weaker in *osddm1b* than in the wild type under BR treatment (Figure 6C), which suggests that *osddm1b* was less sensitive to BRs than the wild type.

### *OsDDM1b* Influences BR Homeostasis and Signaling

According to the above results, loss-of-function of *OsDDM1b* decreased the response to BR treatments, which suggests that *OsDDM1b* may involve in BR signaling. Then, we analyzed the expression levels of BR-related genes, which include *OsBAK1*, *OsBRI1*, *OsBU1*, *OsDLT*, *OsILI1*, *OsLIC*, *OsMDP1*, *OsBZR1*, *OsD2*, and *OsGSK2* in the wild type and *osddm1b* (Yamamuro et al., 2000; Hong et al., 2003; Ito et al., 2005; Duan et al., 2006; Bai et al., 2007; Koh et al., 2007; Wang et al., 2008; Tong et al., 2009, 2012; Zhang et al., 2009). In roots, the *OsBAK1*, *OsBRI1*, *OsILI1*, and *OsBZR1* were slightly upregulated in *osddm1b* (Figure 6D). However, the expression level of *OsBU1* and *OsDLT*, positive regulator of BR signaling, was significantly lower in *osddm1b* than in the wild type. The expression level of *OsLIC*, *OsMDP1*, and *OsGSK2*, as the negative regulator of BR, was also upregulated in *osddm1b*. *OsD2*, as a representative BR biosynthetic gene, was slightly upregulated in *osddm1b*. These results suggested that *OsDDM1b* influenced BR homeostasis and signaling.

It had been reported that the regulation of feedback-inhibited genes in BR biosynthesis usually defects mainly in BR signaling in mutants (Bai et al., 2007; Chen et al., 2013). To confirm this, we analyzed the expression of *OsBRI1*, *OsCPD*, *OsD11*, and *OsDWARF* (Yamamuro et al., 2000; Hong et al., 2002; Tanabe et al., 2005) genes in the wild type and *osddm1b* in the absence or presence of 24-epiBL. In *osddm1b*, BR’s inhibition of these feedback-inhibited genes was released, and they were all strongly suppressed in the mutant than wild type (Figure 6E). To find the essential answers of *OsDDM1b* in regulating BR biosynthesis, the amounts of endogenous BRs were measured in the mutants and wild type (Supplementary Figure 6B). The contents of endogenous BRs were increased by 36.01% in *osddm1b* compared with wild type, which indicated that *OsDDM1b* was required for the feedback inhibition of BR biosynthetic and signaling genes.

### The Effect of Osddm1b on Grain Size-Related Genes

**FIGURE 7:**
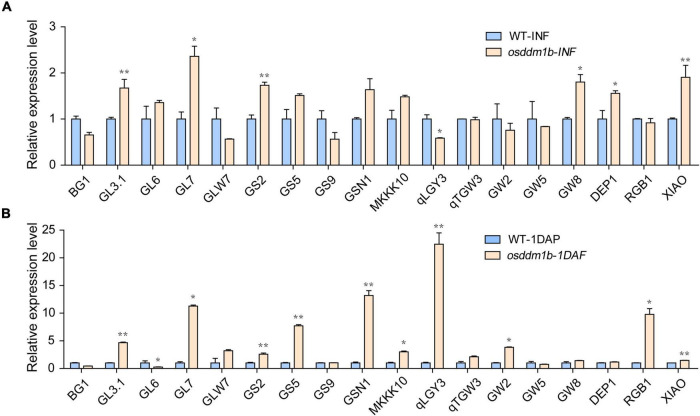
Comparative expression of the genes to determine grain size between wild-type (HY) and *osddm1b* plants. **(A)** The expression level of the genes for the determination of grain size by qRT-PCR using 8 cm panicle. **(B)** The relative expression of grain size determination genes in the seeds of one day after fertilization. Data are given as means ± SD. *p < 0.05, **p < 0.01 compared with wild type (HY) using Student’s *t*-test.

Grain size includes length, width, and thickness, as a quantitative trait locus (QTL), which is vital for grain yield and quality. Thus far, multiple QTLs have been cloned and studied, such as *BG1*, *GL3.1*, *GL6*, *GL7*, *GLW7*, *GS2*, *GS5*, *GS9*, *GSN1*, *MKKK10*, *qLGY3*, *qTGW3*, *GW2*, *GW5*, *GW8*, *DEP1*, *RGB1*, and *XIAO*, which contributed to rice yield (Song et al., 2007; Huang et al., 2009; Li et al., 2011; Jiang et al., 2012; Qi et al., 2012; Wang et al., 2012, 2015, 2019; Hu et al., 2015, 2018; Liu L. C. et al., 2015; Si et al., 2016; Liu et al., 2017, 2018; Guo et al., 2018; Xu et al., 2018; Ying et al., 2018; Zhao et al., 2018). Snf2 family genes, as the chromatin remodeling factors, regulate the transcription of the genes, and our data showed that loss-of-function of *OsDDM1b* resulted in abnormal grain size. To analyze the expression level of grain size-related genes in *osddm1b*, we detected several genes in inflorescence (length 8 cm) and seeds (1 DAF) in wild type and *osddm1b* using qRT-PCR. The results showed that *GL3.1*, *GL7*, *GS2*, *GS5*, *GSN1*, *MKKK10*, *GW8*, *DEP1*, *RGB1*, and *XIAO* were upregulated in *osddm1b* compared with wild type in inflorescence or seeds (Figure 7), and *GL3.1*, *GL7*, *GS2*, and *DEP1* were significantly upregulated. It indicates that *OsDDM1b* negatively regulates their expression level, which affects the grain length (Figure 7A). The loss-of-function of *GSN1*, *MKKK10*, and *XIAO* caused small grain size, which was highly expressed in inflorescence and seeds in *osddm1b*, which suggests that *OsDDM1b* might negatively regulate the expression of these genes (Figures 7A,B). The expression of *GS5* and *GW2*, which affects the milk-filling rate in rice, increased significantly in 1 DAF seeds of *osddm1b*. However, in-depth study is required for identifying that the loss-of-function of *OsDDM1b* influences milk-filling rate in rice.

## Discussion

### Snf2 Gene Family in Rice

Snf2, as a sizeable gene family, has conserved domains (SNF2_N, DEAD and Helicase_C, HELICs) as in animal counterparts, which suggests that they may have a similar function to regulate the gene expression. There are 42 proteins in chromDB database that belong to Snf2 family in rice. The proteins of OsCHR723 and OsCHR744 (homologs of MOM1/CHR15 in *Arabidopsis*) do not have the Helicase_C domain, which is not the member of Snf2 family (Hu et al., 2013). Many genes exhibit specific expression patterns at the different developmental stages of various reproductive tissues (inflorescence, stamens, pistils, and seeds). In addition, some genes have been reported to regulate the abiotic or biotic stress responses, such as *OsALT1* (*alkaline tolerance 1*, *OsCHR706*) (Guo et al., 2014) and *OsBRHIS1* (Li et al., 2015). Here, we report the comparative expression profile of 40 Snf2 genes during the reproductive development to enrich the expression patterns and insights into the functions of this family. However, the responses of the Snf2 gene family under abiotic or biotic stresses have not been systematically analyzed.

### The Function of *DDM1* Is Conserved in *Arabidopsis* and Rice

DDM1 is a nucleosome remodeling ATPase and regulates the DNA damage that is critical influence for the chromatin structure in *Arabidopsis.* The RNAi mutation of *DDM1* has a typical phenotype of demethylation (Shaked et al., 2006). The repeated sequence, even a single-copy sequence, is induced toward hypomethylation after repeated self-pollination, which activates the transposons (Kakutani et al., 1996). The mutation of *DDM1* showed reduced fertility only after repeated self-pollination and produced several kinds of developmental abnormalities. *DDM1* is conserved in regulating DNA methylation. For example, *Lsh* (*lymphocyte-specific helicase*), a homolog gene of *DDM1*, causes genomic DNA hypomethylation in the mouse (Dennis et al., 2001). The antisense *OsDDM1* lines exhibit dwarf phenotypes, and some sublines were observed to be progressive loss of fertility even complete infertility. In addition, methylation is severely reduced in antisense *OsDDM1* lines, and reduction is evident in later generations but not in the genetic transformation process. It is still unknown whether the demethylation reduction was mediated by the repression of the *OsDDM1a/OsCHR746*, *OsDDM1b/OsCHR741*, or both of them. Our data showed that these two homologous genes, *OsDDM1a/OsCHR746* and *OsDDM1b/OsCHR741*, had similar expression patterns and slight differences during seed development, which indicates that the two genes might have diverse functions.

### The Phenotype of Osddm1b on Regulating the Grain Size

The dwarfism phenotype was also observed in the T-DNA insertion mutation of *OsDDM1b* (Figures 2B,H,I and Supplementary Figures 2B,C). The male and female gametophyte formation was found to be normal as expected. However, the seed setting rate was partially decreased to 84.39%, which suggests that *OsDDM1b* might play a role in pollen tube growth or embryo development after fertilization (Supplementary Figure 2D–H). The dwarfism and partial fertility are similar to antisense *OsDDM1* lines. The single mutation of *OsDDM1a* or *OsDDM1b* showed a normal phenotype during vegetative or reproductive stages, even in several generations (Tan et al., 2016). However, our data showed that *osddm1b* exhibited dwarfism and grain size variations. The T-DNA insertion in Tan’s mutation of *OsDDM1b* was in the third intron. However, in our experiment, the mutation was in the second exon (Figure 2A and Supplementary Figure 2A, see footnote 3), which suggests that this difference may be due to the position of T-DNA insertion. *OsDDM1a* and *OsDDM1b*, as the homologous genes, have a significantly similar genome and amino acid sequences. The primers of qRT-PCR were designed by aligning the CDS of two genes and finding the different bases among T-DNA insertion positions to improve primers’ specificity and obtain more accurate expression data (Figure 2A).

As an important agronomic trait, grain size is essential for crop genetics and breeding, which includes length, width, and thickness. Several genes have been reported to play the critical roles in regulating grain size. Cell proliferation and expansion are coordinately significant in the spikelet hull, which controls the final grain size. The expression levels of both *OsDDM1* are more enriched in proliferating young tissues (Hu et al., 2013), which is consistent with the expression pattern of *OsDDM1b* by GUS staining (Figures 5B,C and Supplementary Figure 5). The results of the paraffin section and SEM suggested that *OsDDM1b* might promote latitudinal growth by increasing cell proliferation to contribute to the grain size (Figure 4 and Supplementary Figure 4). Many regulators are involved in controlling grain size, such as G protein signaling (*DEP1*, *RGB1*) (Huang et al., 2009; Zhang et al., 2021), the ubiquitin–proteasome pathway (*GW2*) (Song et al., 2007), the MAPK signaling pathway (*GSN1*, *MKKK10*, and *MAPK6*) (Liu S. Y. et al., 2015; Guo et al., 2018; Xu et al., 2018), phytohormone signaling (*BG1*, *GL3*.1, and *GS5*) (Li et al., 2011; Qi et al., 2012; Liu L. C. et al., 2015), transcriptional regulation (*GL7*, *GLW7*/*OsSPL13*, *GS2*/*OsGRF4*, *GS9*, *GW8*/*OsSPL16*, and *qLGY3*/*OsMADS1*) (Wang et al., 2012, 2015; Hu et al., 2015; Si et al., 2016; Liu et al., 2018; Zhao et al., 2018), and other regulators such as protein kinase (*qTGW3*/*OsGSK5*) (Hu et al., 2018). The creation of insertion mutations is a valuable tool for active transposons to analyze the function of endogenous genes (Hirochika, 2001). The abnormal grain size was caused by the defection of *OsDDM1b*, which affected the expression of grain size regulators.

There may be a balance mechanism in plants when the mutation of *OsDDM1b* displayed decreased grain length. In contrast, grain width and thickness were increased due to the compensation mechanism or regulated grain filling rate to balance the 1,000-grain weight with wild type. The higher expression levels of *GS5* produced more comprehensive grains and increased grain filling rate. Then, *GS5* was upregulated in the inflorescence (length 8 cm) and seeds (1 DAF) of *osddm1b*, which suggests that *OsDDM1b* might negatively regulate *GS5* to affect the grain width. However, whether *OsDDM1b* can influence the grain filling rate is still unknown.

### A Proposed Model for *OsDDM1b* Regulation on Grain Size

Above all, *OsDDM1b* (decrease in DNA methylation) contributes to grain size by regulating latitudinal cell proliferation and cell width to enhance the grain width and thickness, which decreases the grain length reversely. In addition, *OsDDM1b*, as a chromatin remodeling regulator, influences BR homeostasis and signaling pathway, the expression levels of BR, and grain size-related genes (Supplementary Figure 7). DNA methylation is involved in embryo and endosperm development and is required to determine the methylation pattern in *osddm1b*. The relationship between grain size and dynamic DNA methylation is necessary to be revealed with the future studies. This helps to understand the mechanism of epigenetic regulation in controlling grain size in rice.

## Data Availability Statement

The original contributions presented in the study are included in the article/Supplementary Material, further inquiries can be directed to the corresponding authors.

## Author Contributions

YQ and HZ conceived the initial screening and research plans and designed the experiments. MG, WZ, ZH, ZS, MY, CS, LL, AW, and JL performed most of the experiments. ZS, MY, CS, LL, and AW extracted the RNA. MM, MG, and WZ performed the qRT-PCR and analyzed the data. ZH and DT performed the paraffin section and SEM. YQ, HZ, and MG wrote the article with the contributions of all the authors. All authors contributed to the article and approved the submitted version.

## Conflict of Interest

The authors declare that the research was conducted in the absence of any commercial or financial relationships that could be construed as a potential conflict of interest.

## Publisher’s Note

All claims expressed in this article are solely those of the authors and do not necessarily represent those of their affiliated organizations, or those of the publisher, the editors and the reviewers. Any product that may be evaluated in this article, or claim that may be made by its manufacturer, is not guaranteed or endorsed by the publisher.

## Footnotes

1
http://gsds.gao-lab.org/index.php

2
https://imagej.nih.gov/ij/

3
http://signal.salk.edu/cgi-bin/RiceGE

## Supplementary Material

Supplementary Figure 1 The relative expression level of Snf2 gene family in the wild-type (ZH11) seeds. **(A)** Highly expressed *OsCHR714* in the S3 (7 DAF) stage, while *OsCHR728* is just lowly expressed in the S5 (25 DAF) stage. **(B)** Preferentially expressed *OsCHR720*, *726*, and *740* in the S4 (10 DAF) stage. **(C)** predominately expressed *OsCHR717*, *724*, *743*, and *746* in the S4 (10 DAF) or S5 (25 DAF) stage. S1, the seeds of 1 DAF; S2, the seeds of 3 DAF; S3, the seeds of 7 DAF; S4, the seeds of 10 DAF; S5, the seeds of 25 DAF.

Supplementary Figure 2 Genotype and phenotype identification of *OsDDM1b* mutant. **(A)** The young seedlings of wild type (HY) and *osddm1b* in 10 days. Bar = 4 cm. **(B)** The internode length of wild type (HY) and *osddm1b*. Bar = 5 cm. **(C,D)** Viability of mature pollen grains in wild type **(C)**, (HY) and *osddm1b* **(D)** as assessed by I_2_-KI staining. Bar = 200 μm. **(E,F)** Mature ovules in the wild type **(E)**, (HY) and *osddm1b* **(F)**. Bar = 25 μm. **(G)** The seed setting rate of wild type (HY) and *osddm1b* (*n* = 10). **(H)** The 1,000-grain weight of wild type (HY) and *osddm1b* (*n* = 27). **(I–N)** Comparation on grain length **(I,J)**, width **(K,M)**, and thickness **(L,N)** of wild type (HY) and *osddm1b*. **(G–H)** Bar = 5 mm. **(I–L)** Bar = 2 mm. Data are given as means ± SD. ^NS^*p* > 0.05, NS: not significant.

Supplementary Figure 3 Identification of T-DNA insertion site. A segment was amplified from *osddm1b* homozygous mutants DNA using the 2B-60109-Lp and Rb (2707) primers. The PCR fragments were sequenced and aligned with the genome of *OsDDM1b* and 2707 vector sequence by the SnapGene software, respectively.

Supplementary Figure 4 Histological analysis of spikelet hulls and the effect of *OsDDM1b* on the expression of genes involved in the cell cycle. **(A,B)** Magnified views of the cross-section of wild type (HY) and *osddm1b*. The black and blue arrows indicate the rows of specialized cells with lower epidermis cells and rigid walls, respectively. Bar = 50 μm. **(C)** The expression levels of cell cycle-related genes in the 8-cm panicles. Data are given as means ± SD. ***p* < 0.01, **p* < 0.05 compared with wild type (HY) using Student’s *t*-test.

Supplementary Figure 5 The native *OsDDM1b* promoter drives GUS expression. **(A–D)** GUS signal was detected in young root tips **(A)**, young leaf **(B)**, mature leaf **(C)**, stem node **(D)**. Bar = 1 mm **(A)**, Bar = 2 mm **(B–D)**. **(E–F)** The stamen **(E)** and pistil **(F)** from 1–2, 2–3, 3–4, 4–5, 5–6, 6–7 cm, and mature spikelet, respectively. Bar = 500 μm.

Supplementary Figure 6 The phenotype of wild type (HY) and *osddm1b* in response to BR treatment. **(A)** Lamina joint of the wild type (HY) and *osddm1b* in response to various concentrations of BR. Bar = 2 mm. **(B)** The contents of endogenous BR in the wild type (HY) and *osddm1b*. Data are given as means ± SD. ***p* < 0.01 compared with wild type (HY) using Student’s *t*-test.

Supplementary Figure 7 A working model on *OsDDM1b* regulation on grain size. As an epigenetic regulator, *OsDDM1b* is involved in DNA methylation, which participates in multiple biological processes. For example, *OsDDM1b* affected the expression of key genes determining cell cycle, BR signal, and grain size. Abnormal cell proliferation can influence the character of spikelets resulting in grain size defection.
