# Supplementary figures and images for: OsDDM1b Controls Grain Size by Influencing Cell Cycling and Regulating Homeostasis and Signaling of Brassinosteroid in Rice
Source: Front Plant Sci. 2022 Apr 8;13:873993. doi: 10.3389/fpls.2022.873993 (PMC9024357; doi:10.3389/fpls.2022.873993)

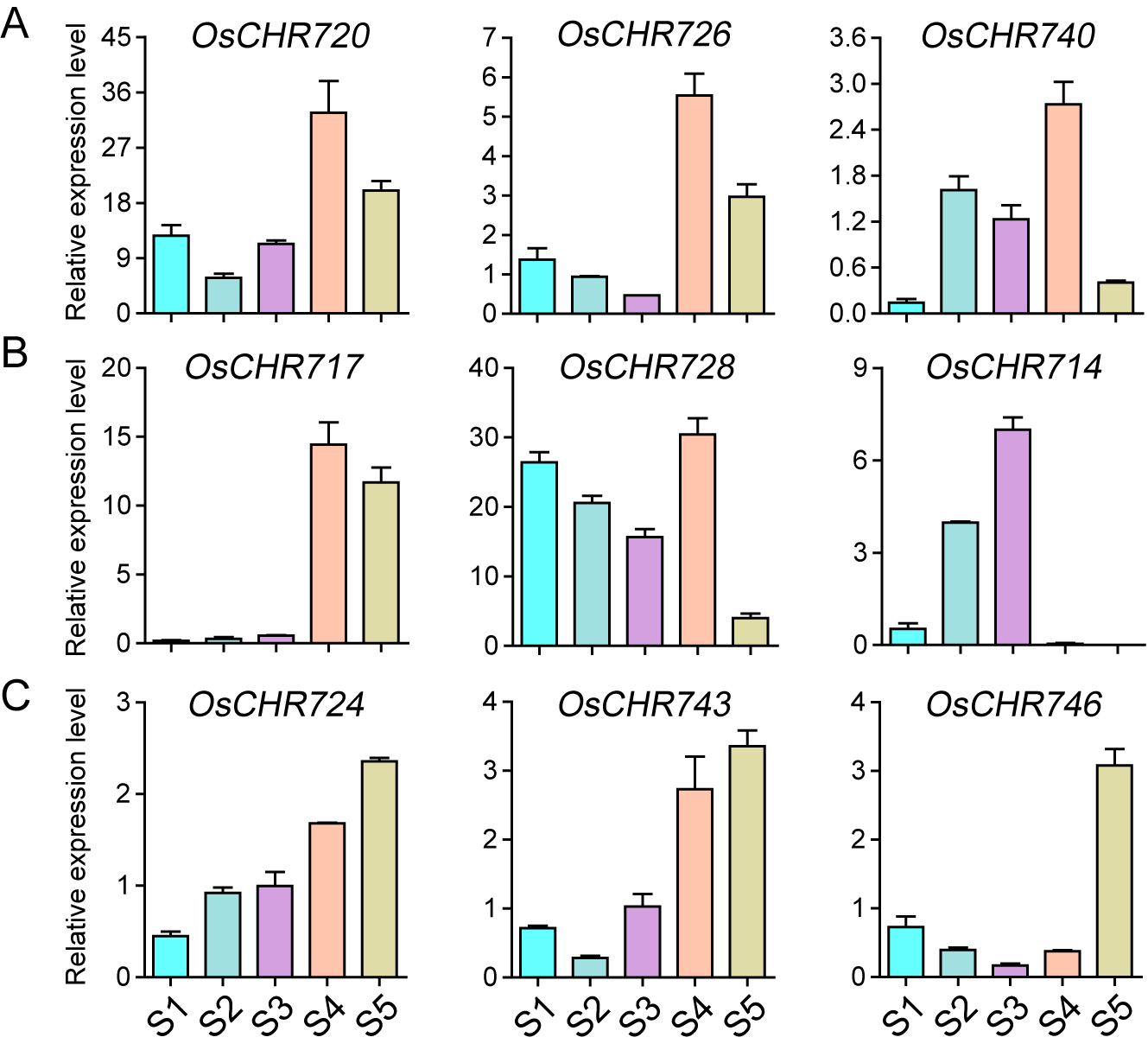

Supplement: Supplementary Figure 1 — The relative expression level of Snf2 gene family in the wild-type (ZH11) seeds. (A) Highly expressed OsCHR714 in the S3 (7 DAF) stage, while OsCHR728 is just lowly expressed in the S5 (25 DAF) stage. (B) Preferentially expressed OsCHR720, 726, and 740 in the S4 (10 DAF) stage. (C) predominately expressed OsCHR717, 724, 743, and 746 in the S4 (10 DAF) or S5 (25 DAF) stage. S1, the seeds of 1 DAF; S2, the seeds of 3 DAF; S3, the seeds of 7 DAF; S4, the seeds of 10 DAF; S5, the seeds of 25 DAF. [file Image_1.TIF]

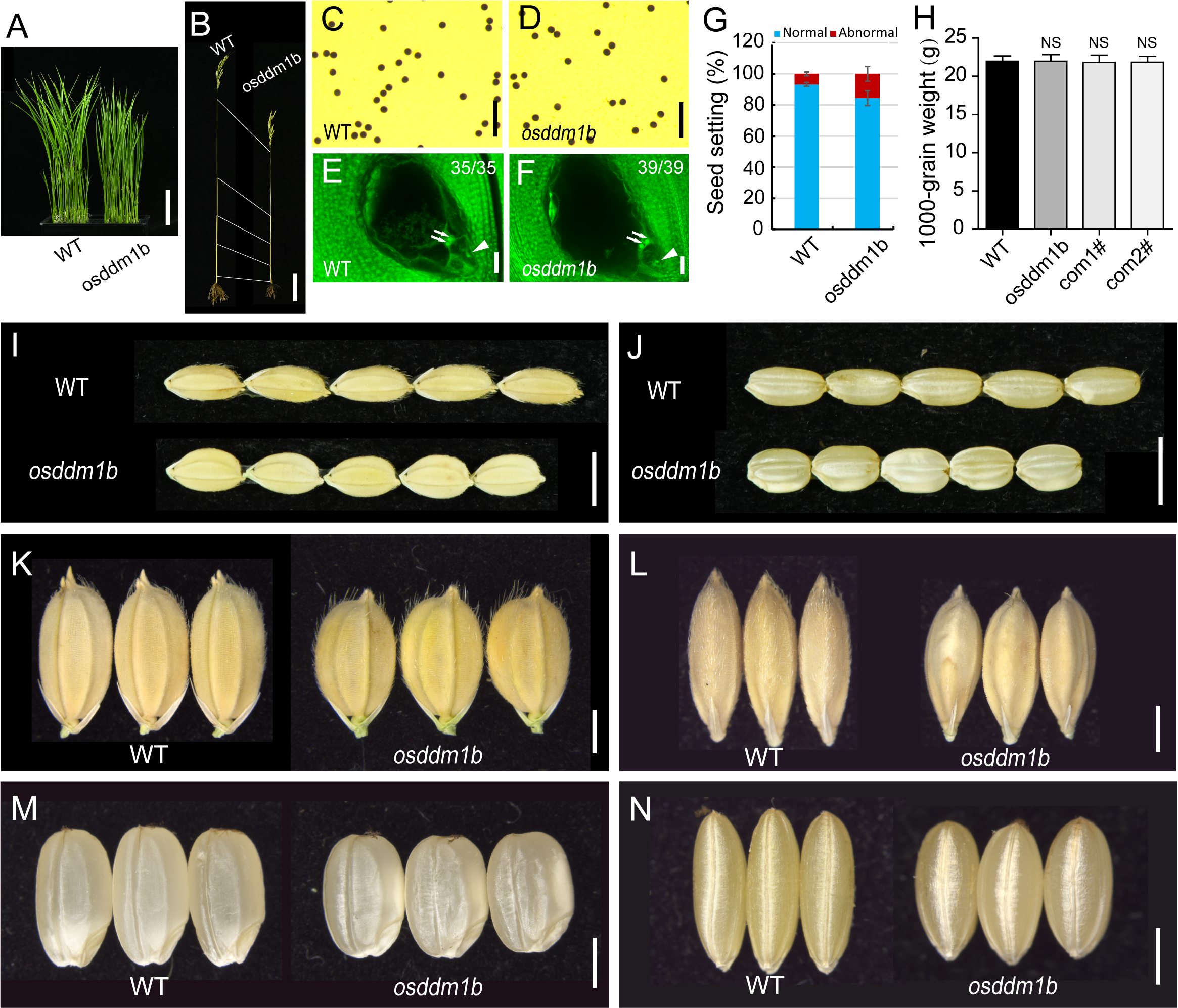

Supplement: Supplementary Figure 2 — Genotype and phenotype identification of OsDDM1b mutant. (A) The young seedlings of wild type (HY) and osddm1b in 10 days. Bar = 4 cm. (B) The internode length of wild type (HY) and osddm1b. Bar = 5 cm. (C,D) Viability of mature pollen grains in wild type (C), (HY) and osddm1b (D) as assessed by I2-KI staining. Bar = 200 μm. (E,F) Mature ovules in the wild type (E), (HY) and osddm1b (F). Bar = 25 μm. (G) The seed setting rate of wild type (HY) and osddm1b (n = 10). (H) The 1,000-grain weight of wild type (HY) and osddm1b (n = 27). (I–N) Comparation on grain length (I,J), width (K,M), and thickness (L,N) of wild type (HY) and osddm1b. (G–H) Bar = 5 mm. (I–L) Bar = 2 mm. Data are given as means ± SD. NSp > 0.05, NS: not significant. [file Image_2.TIF]

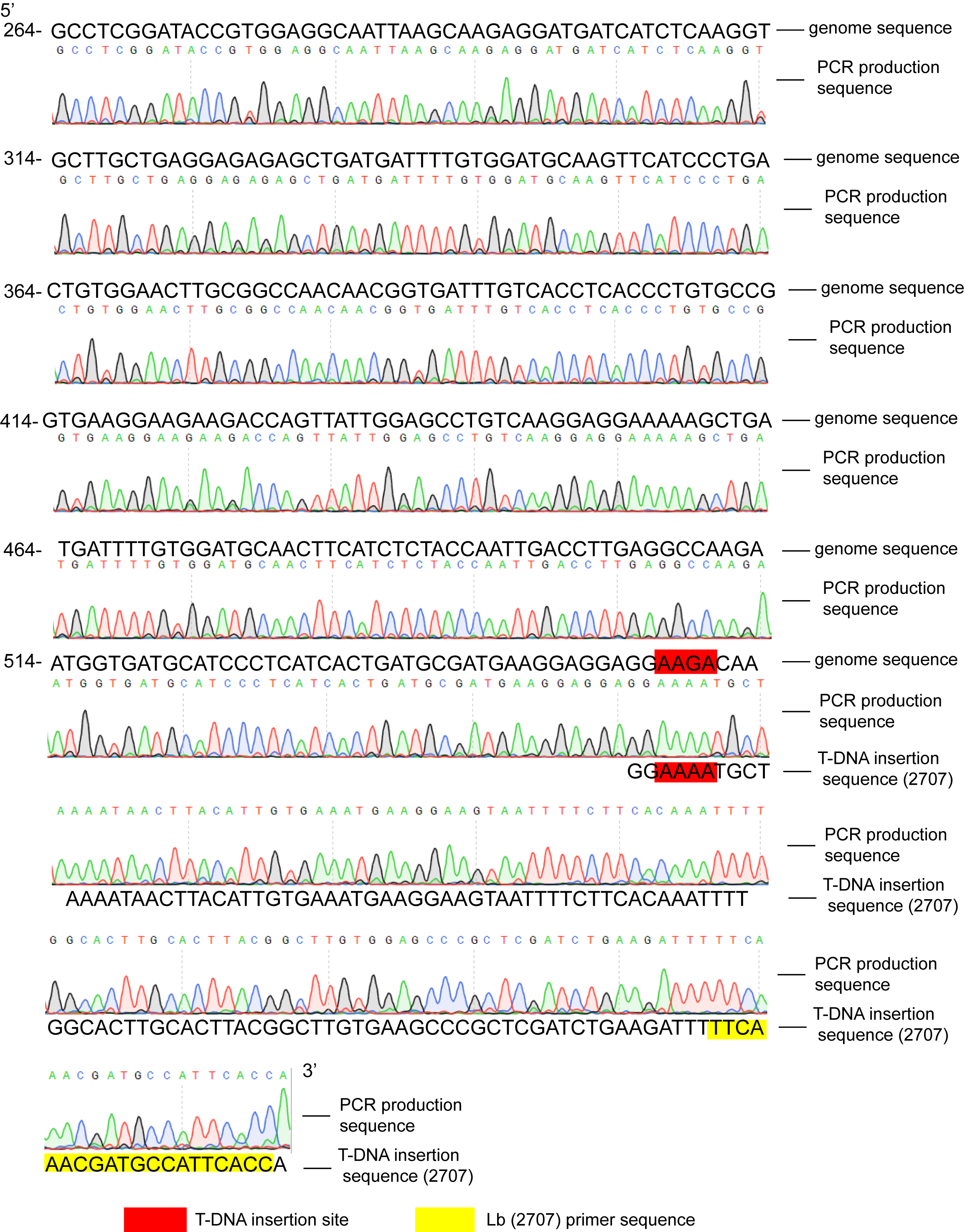

Supplement: Supplementary Figure 3 — Identification of T-DNA insertion site. A segment was amplified from osddm1b homozygous mutants DNA using the 2B-60109-Lp and Rb (2707) primers. The PCR fragments were sequenced and aligned with the genome of OsDDM1b and 2707 vector sequence by the SnapGene software, respectively. [file Image_3.TIF]

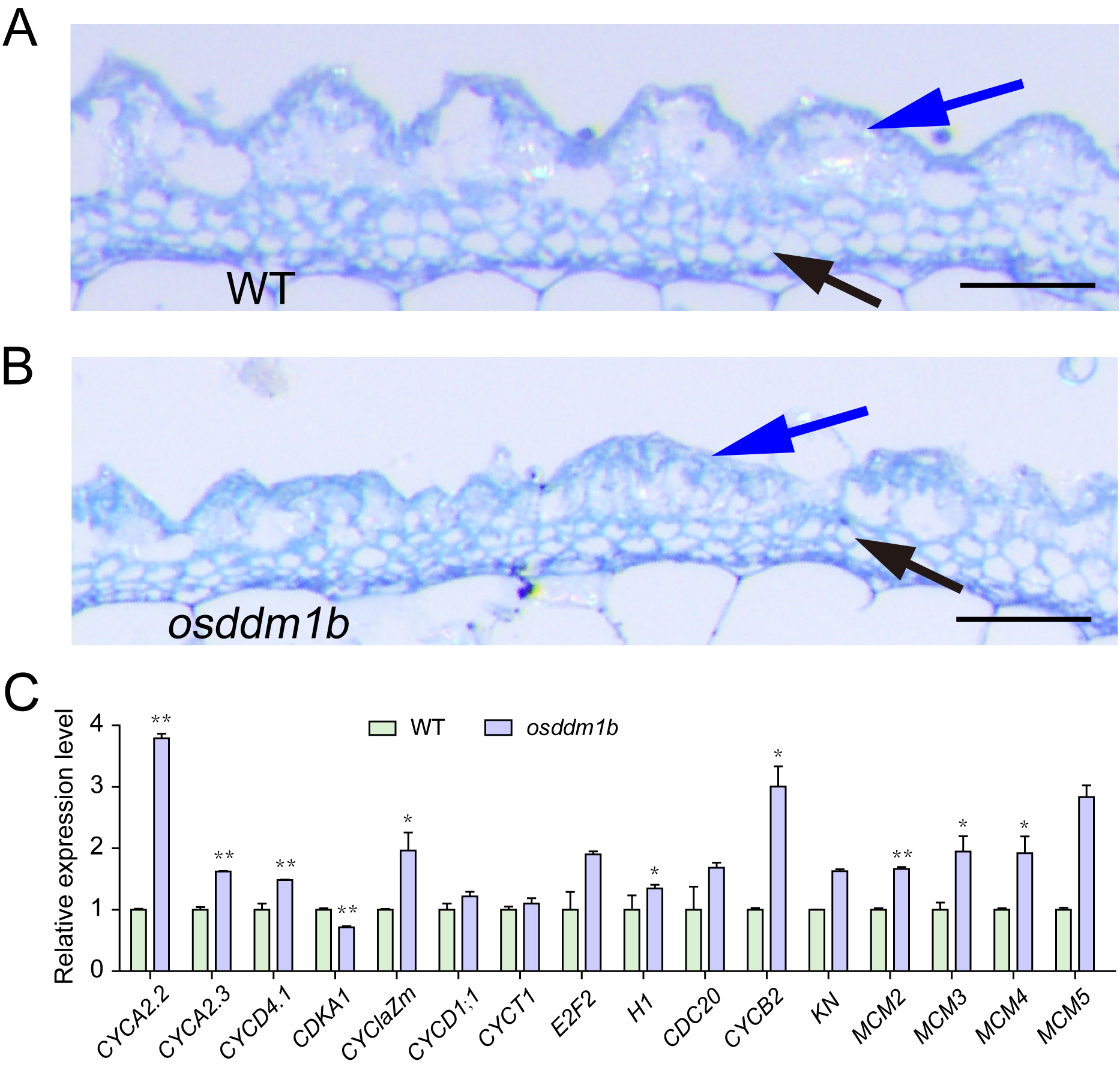

Supplement: Supplementary Figure 4 — Histological analysis of spikelet hulls and the effect of OsDDM1b on the expression of genes involved in the cell cycle. (A,B) Magnified views of the cross-section of wild type (HY) and osddm1b. The black and blue arrows indicate the rows of specialized cells with lower epidermis cells and rigid walls, respectively. Bar = 50 μm. (C) The expression levels of cell cycle-related genes in the 8-cm panicles. Data are given as means ± SD. **p < 0.01, *p < 0.05 compared with wild type (HY) using Student’s t-test. [file Image_4.TIF]

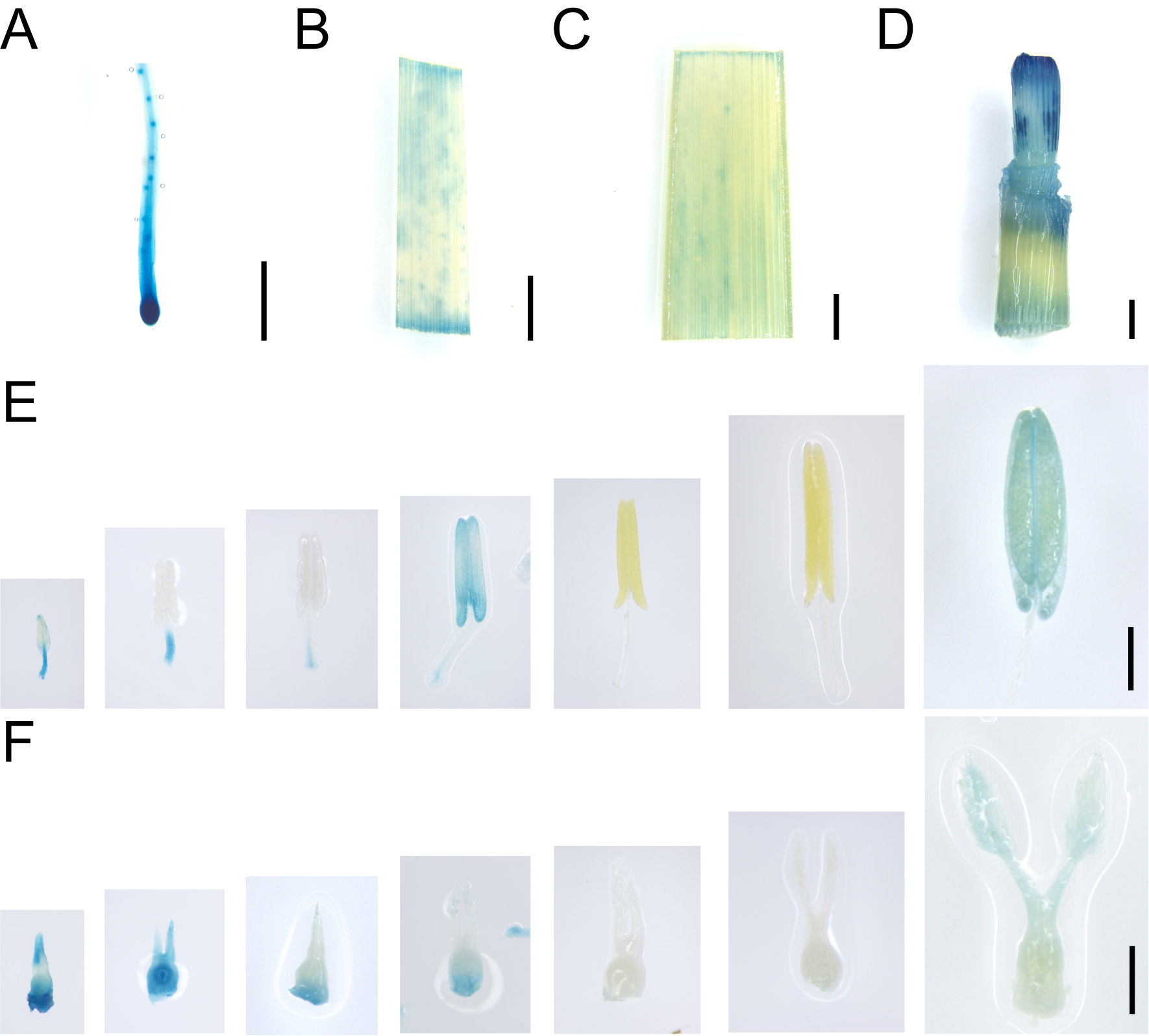

Supplement: Supplementary Figure 5 — The native OsDDM1b promoter drives GUS expression. (A–D) GUS signal was detected in young root tips (A), young leaf (B), mature leaf (C), stem node (D). Bar = 1 mm (A), Bar = 2 mm (B–D). (E–F) The stamen (E) and pistil (F) from 1–2, 2–3, 3–4, 4–5, 5–6, 6–7 cm, and mature spikelet, respectively. Bar = 500 μm. [file Image_5.TIF]

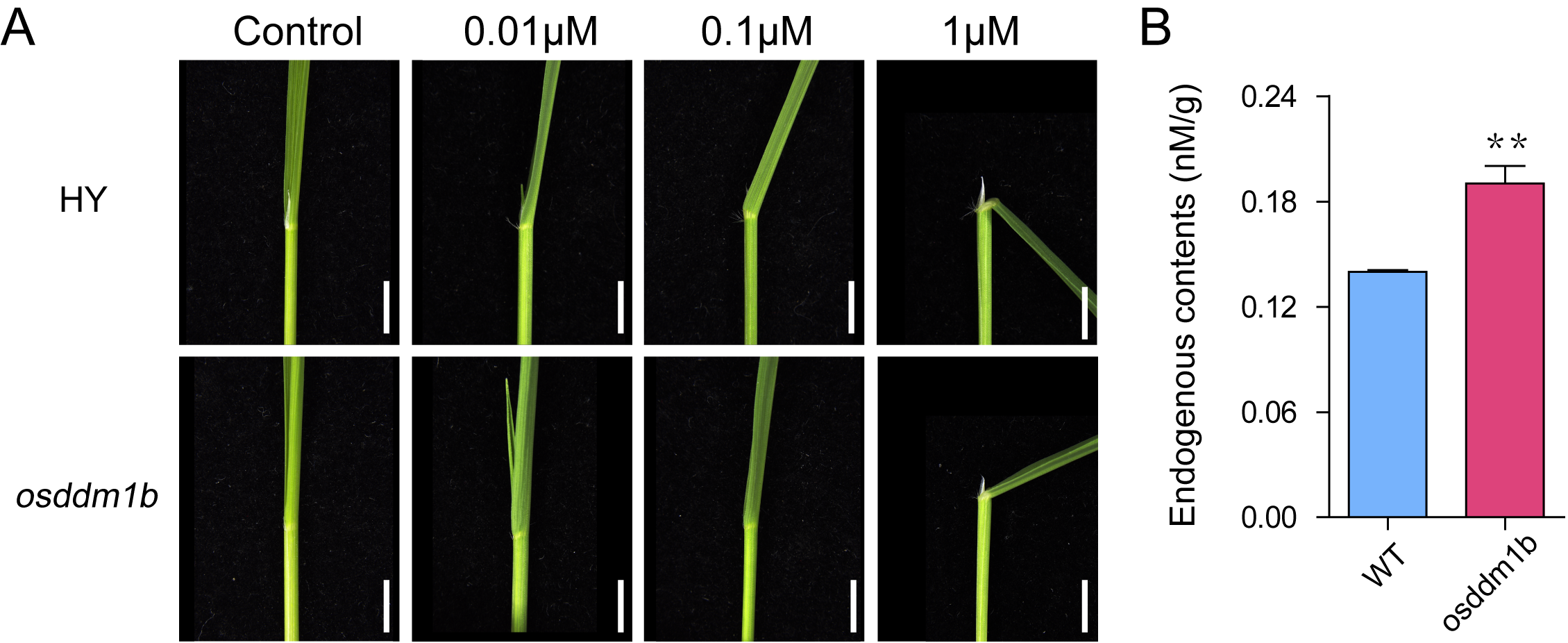

Supplement: Supplementary Figure 6 — The phenotype of wild type (HY) and osddm1b in response to BR treatment. (A) Lamina joint of the wild type (HY) and osddm1b in response to various concentrations of BR. Bar = 2 mm. (B) The contents of endogenous BR in the wild type (HY) and osddm1b. Data are given as means ± SD. **p < 0.01 compared with wild type (HY) using Student’s t-test. [file Image_6.TIF]

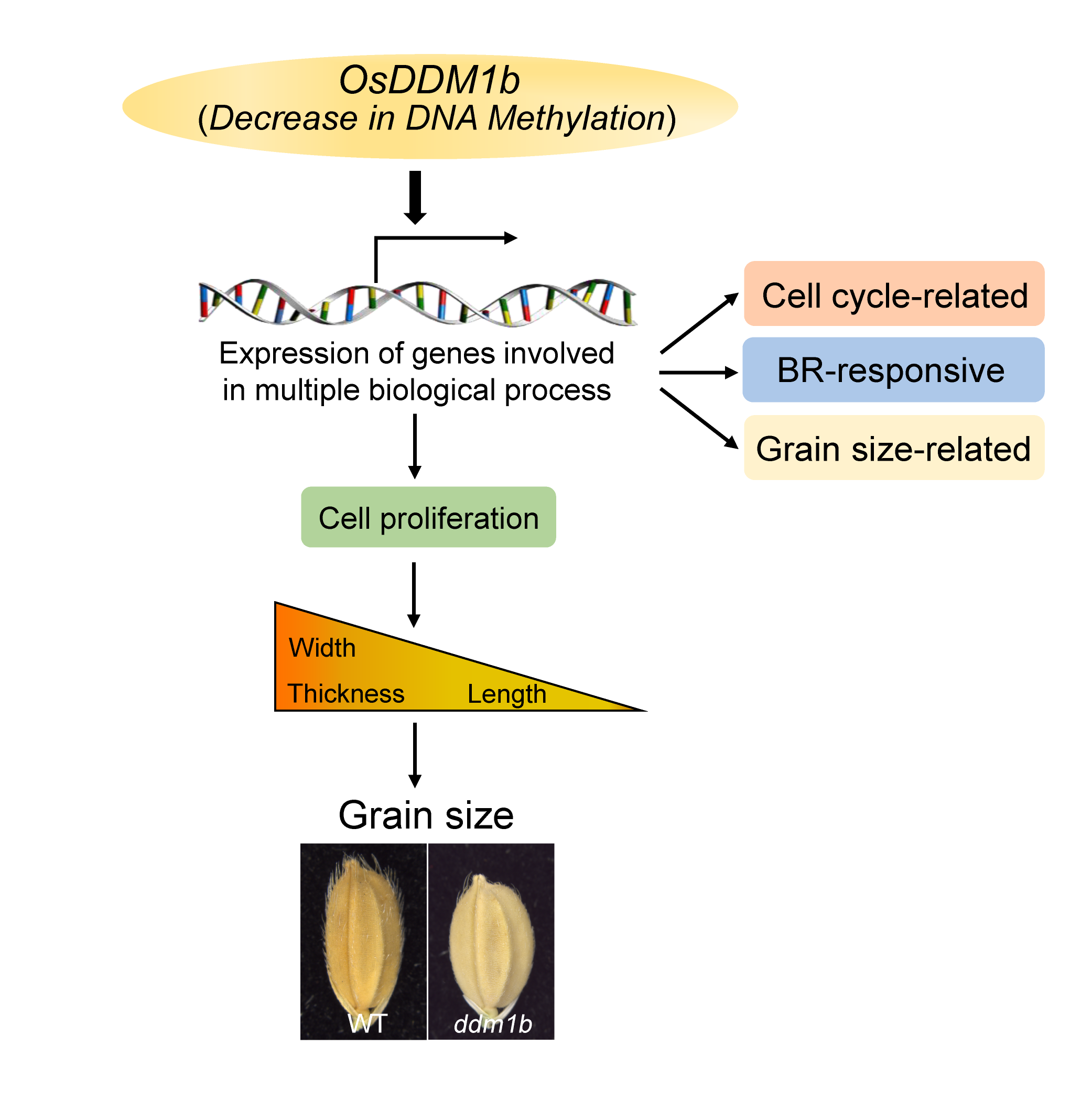

Supplement: Supplementary Figure 7 — A working model on OsDDM1b regulation on grain size. As an epigenetic regulator, OsDDM1b is involved in DNA methylation, which participates in multiple biological processes. For example, OsDDM1b affected the expression of key genes determining cell cycle, BR signal, and grain size. Abnormal cell proliferation can influence the character of spikelets resulting in grain size defection. [file Image_7.TIF]
